# Spatiotemporal monitoring of climate change impacts on water resources using an integrated approach of remote sensing and Google Earth Engine

**DOI:** 10.1038/s41598-024-56160-9

**Published:** 2024-03-05

**Authors:** Mohammad Kazemi Garajeh, Fatemeh Haji, Mahsa Tohidfar, Amin Sadeqi, Reyhaneh Ahmadi, Narges Kariminejad

**Affiliations:** 1 Department of Civil, Constructional and Environmental Engineering, Sapienza University of Rome, 00185 Rome, Italy; 2 School of Engineering, Università degli Studi della Basilicata, Via Nazario Sauro 85, 85100 Potenza, Italy; 3 Department of Earth Sciences, Remote Sensing and GIS, Shahid Beheshti University, Tehran, Iran; 4 Department of Natural Resources and Environment, Islamic Azad University, Science and Research Branch, Tehran, Iran; 5 Department of Geography and Geology, University of Turku, 20014 Turku, Finland; 6 Department of Regional and City Planning, Christopher C. Gibbs College of Architecture, University of Oklahoma, Norman, USA; 7 Department of Natural Resources and Environmental Engineering, College of Agriculture, Shiraz University, Shiraz, Iran

**Keywords:** Climate change, Water resources, Google Earth Engine, Remote sensing, Time series analysis, Climate sciences, Environmental sciences, Environmental social sciences, Hydrology, Natural hazards, Planetary science

## Abstract

In this study, a data-driven approach employed by utilizing the product called JRC-Global surface water mapping layers V1.4 on the Google Earth Engine (GEE) to map and monitor the effects of climate change on surface water resources. Key climatic variables affecting water bodies, including air temperature (AT), actual evapotranspiration (ETa), and total precipitation, were analyzed from 2000 to 2021 using the temperature-vegetation index (TVX) and Moderate Resolution Imaging Spectroradiometer (MODIS) products. The findings demonstrate a clear association between global warming and the shrinking of surface water resources in the LUB. According to the results, an increase in AT corresponded to a decrease in water surface area, highlighting the significant influence of AT and ETa on controlling the water surface in the LUB (partial rho of − 0.65 and − 0.68, respectively). Conversely, no significant relationship was found with precipitation and water surface area (partial rho of + 0.25). Notably, the results of the study indicate that over the past four decades, approximately 40% of the water bodies in the LUB remained permanent. This suggests a loss of around 30% of the permanent water resources, which have transitioned into seasonal water bodies, accounting for nearly 13% of the total. This research provides a comprehensive framework for monitoring surface water resource variations and assessing the impact of climate change on water resources. It aids in the development of sustainable water management strategies and plans, supporting the preservation and effective use of water resources.

## Introduction

Population growth, climate change, and globalization are the main factors affecting water resources^1^. Among the main controlling factors, climate changes play a serious role when researchers evaluate both the quality and quantity of water resources^2,3^. Climate change is already affecting water access for people around the world, causing more severe droughts and floods. It impacts the water cycle by influencing when, where, and how much precipitation falls^4,5^. Climate change also profoundly effects water resources by shifting rainfall patterns, enhancing temperatures, and altering the timing of glacier melt and snowfall, leading to changes in the seasonality of drainage flows^6,7^. Therefore, identifying various predisposing climatic variables is a crucial issue for determining the impacts of climate change on water resources^8,9^. Meteorological datasets are the most common way to monitor the effects of climate change. However, traditional mapping based on meteorological stations is labor-intensive, slower, and more expensive. Additionally, the problems with sensors placed there can increase the error in the obtained data^10^. In this regard, during the last decades, remote sensing technology has been exhibiting high potential for monitoring the impacts of climate change at large scales^11–13^. One of the main benefits of remote sensing lies in its non-intrusive nature^14^. Passive sensors capture electromagnetic energy without disrupting the object or area under observation. This enables researchers to monitor natural phenomena without requiring changes to their methods or behaviors^15^. Remote sensing also offers valuable data on critical climate variables such as temperature, precipitation, and vegetation dynamics. By conducting ongoing monitoring, scientists can analyze patterns, simulate climate scenarios, and assess the effects of climate change on ecosystems and susceptible regions^16^.

In recent decades, Lake Urmia (LU) has experienced significant desiccation, primarily due to inadequate water management. The construction of numerous upstream dam reservoirs, which has led to the expansion of irrigated agriculture, is the main culprit behind this imposed disaster. Irrigated agriculture stands out as the largest water user in the region^17,18^. LU has been shrinking at an alarming rate. Between 1996 and 2016, its water level dropped by approximately 8 m^19^. This drastic reduction in the lake's size has not only posed a threat to its valuable ecosystem and the local economy but has also resulted in severe problems such as dust storms and skin and respiratory diseases^20,21^. Efforts have been made to identify the factors contributing to this alarming shrinkage of the LU. During the period of the lake's decline, there has been an increasing trend in temperature and a decreasing trend in precipitation within the Lake Urmia Basin (LUB)^22–25^. Additionally, agricultural land has expanded by 98% from 1987 to 2016^19^, leading to a significant increase in water withdrawal from rivers and groundwater resources during the same period^22^. Consequently, the inflow of water into the lake has decreased^26^. Thus, the alarming shrinkage of the lake can be attributed to a combination of climatic extremes and anthropogenic activities, along with unsustainable water management^27^. To address the environmental tragedy of the LU, the Lake Urmia Restoration Program (ULRP) was initiated in 2013 as a ten-year program. The ULRP is divided into three phases: (i) stabilizing the current status (2014–2016), (ii) restoration (2016–2020), and (iii) sustainable restoration (2020–2024). Several studies have assessed the progress of the ULRP, highlighting various challenges. Danesh-Yazdi and Ataie-Ashtiani^28^ have raised concerns about the underestimation of data and the importance of data-driven modeling in the ULRP. Sima et al.^29^ noted that the ecological level set by the ULRP (1274.1 m above sea level) falls short of achieving the target salinity of 240 g/l. Saemian et al.^30^ concluded that 80% of the increase in the lake's water volume is attributed to increased inflow from rivers and reservoirs, and recovery may be compromised during prolonged drought periods. Parsinejad et al.^31^ argued that lake restoration requires a comprehensive approach that considers both human and natural components.

The impacts of climate oscillations and climate change on the occurrence of surface and subsurface water can be calculated and analyzed to demonstrate how water resources are affected by natural or human activities^32^. Global datasets indicating water resource locations have been obtained from satellite imagery observational data, inventories, and national descriptions. However, investigating climate changes at both spatial and temporal scales remains challenging^33,34^. Many studies focus on measuring present or future water resources^35–38^ and water quality^39–42^ using information derived from satellite imagery. These studies contribute to the development of sustainable water management approaches by evaluating the impacts of climate change and global warming on water resources. Global datasets documenting the location and seasonal variations of surface water have been generated through inventories, national reports, statistical extrapolation from regional data, and satellite imagery. However, accurately measuring long-term changes at high resolution remains a challenging task. Although remote sensing-based methods and products have employed for mapping water resources in water studies, it is important to consider new products such as JRC Global Surface Water Mapping Layers, v1.4 with a spatial resolution of 30 m to update these datasets and approaches. It furnishes valuable statistics pertaining to the extent and alterations in water surfaces^36^. This product offers comprehensive insights into various aspects of water surfaces, including seasonal, temporal, monthly, and permanent reservoir information. It distinguishes itself from other Landsat products, most notably the Global Land Analysis and Discovery (GLAD) dataset spanning from 1999 to 2020^43^, the global surface water dataset (GSWD) covering the years 1984 to 2015^36^, and the landsat dynamic surface water extent (DSWE) dataset developed by^44^.

The literature review reveals that several studies have utilized remote sensing datasets in combination with the Google Earth Engine (GEE) platform to map and monitor long-term changes in water surfaces^45–47^. Several studies^48–50^ have also explored the relationship between fundamental climatic variables (such as air temperature (AT), evapotranspiration (Eta), and precipitation) and water resources. Previous studies have employed the GEE platform to map water surface changes using Sentinel and Landsat series images^45,47,51^. However, limited research has utilized the Landsat high-resolution mapping of the global surface water product (JRC Global Surface Water Mapping Layers, v1.4) for mapping and monitoring changes in water resources^36,52^. Therefore, the objectives of this study are to: (1) monitor the impacts of climate change on water resources in LUB, Iran, (2) estimate the relationship between different climatic variables (AT, ETa, and precipitation) and changes in water surfaces, and (3) assess the efficiency of the Landsat high-resolution surface water product in tracking trends in various water resources.

### Study area

**Figure 1:**
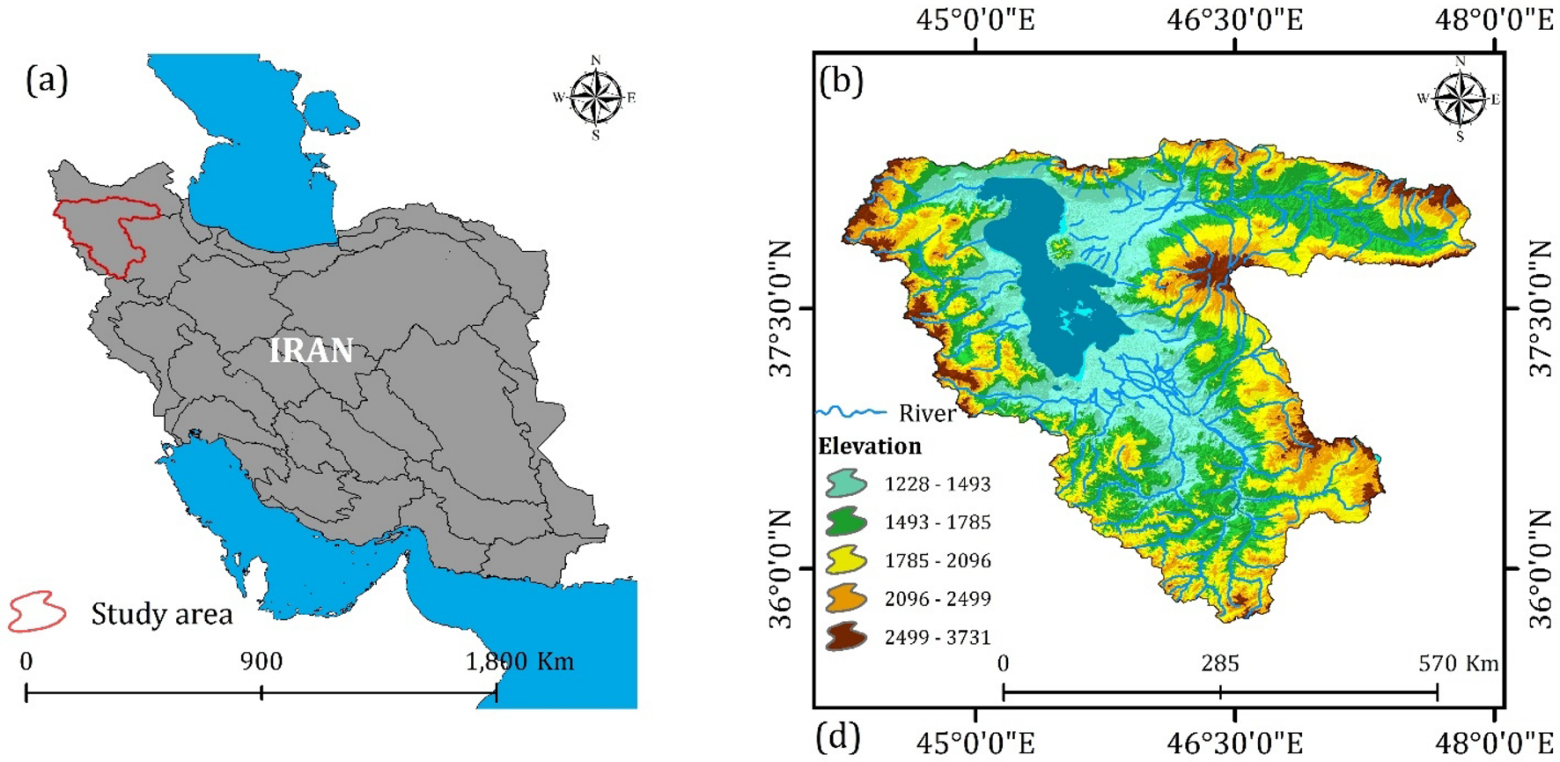
Maps of the LUB, generated in the ArcGIS 10.7.1 software (www.esri.com), depicting: (**a**) its location within Iran's basins, and (**b**) a hydro-topographic representation.

The study area is the LUB, located in the northeast of Iran, with a land area of 51,876 km^2^ (Fig. 1). Approximately 10% of this area is occupied by LU. The LUB is fed by a total of 60 rivers, of which 21 are permanent or seasonal, and 39 are temporary. Notably, the Zarineh river, Simineh river, and Aji Chai serve as the main inflows to LU. Within this basin, Zarineh river (14%), Simineh river (11%), Godar (8%), Barandoz (6%), Shahrchai (2%), and Nazlu Chai (6%) are joined by seven seasonal rivers, 39 intermittent streams, internal springs, as well as rainfall and snowfall to sustain LU. The rivers in this watershed are categorized based on their water sources, exhibiting distinct characteristics in the east and west of the lake. The rivers to the east and southeast of the lake originate from high-altitude sources such as Sahand, Sabalan, and Chehel Cheshme in Kurdistan, following relatively longer courses and remaining perennial (e.g., Aji Chai, Zarineh river, Simineh river, and Sufi Chai). In contrast, the western, southwestern, and northern rivers of the lake, which have shorter distances and carry less water, include Godar Chai, Nazlo Chai, and Zola Chai^22,53^. The region is characterized by a semi-arid and dry-cold climate, with an average annual rainfall of 359.1 mm, a minimum temperature of 4.3 °C, and a maximum temperature of 17.7 °C^54^. Evaporation rates range from 930 to 1513 mm per year^55^. The Lake receives approximately 25% of its inflow from direct precipitation and 75% from 60 permanent or seasonal rivers^56^.

**Figure 2:**
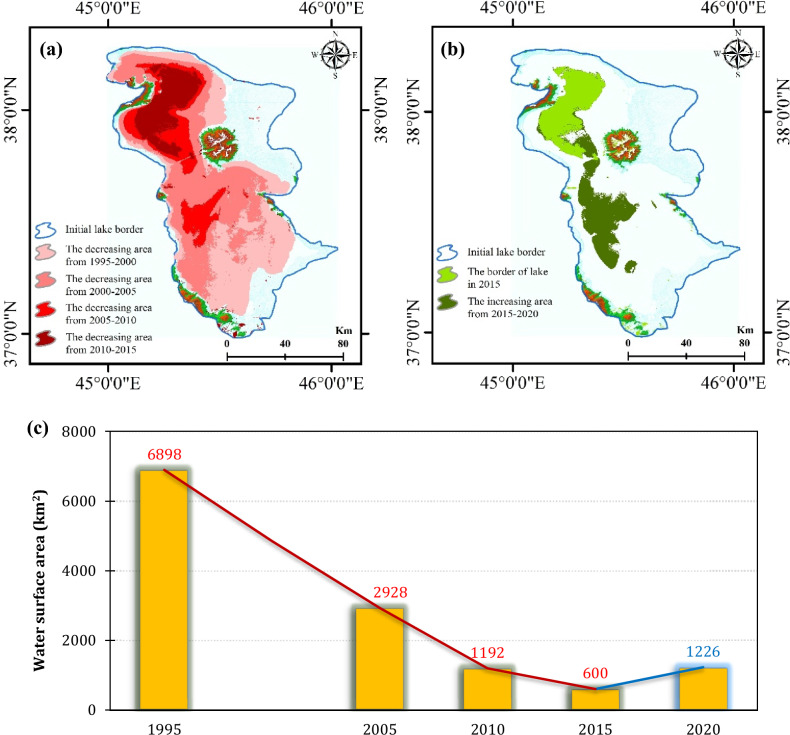
Spatio-temporal changes in water surface areas of Lake Urmia: (**a**) spatial decline in water surface areas from 1995 to 2015, (**b**) spatial increase in water surface areas from 2015 to 2020, generated in the ArcGIS 10.7.1 software (www.esri.com, and (**c**) temporal variation chart of water surface areas, generated in the Microsoft Office Excel 2023 (https://www.microsoft.com).

There have been significant changes in the water surface area of the LU from year to year (Fig. 2), particularly in the southern regions of the lake where the lake bed occasionally becomes exposed during summer and fall^57^. From 2014 to 2016, 2015 experienced the minimum lake surface area, while 2016 experienced the maximum lake surface area. In August, the minimum and maximum surface areas were 1550 km^2^ and 2500 km^2^ in 2015 and 2016, respectively, clearly indicating the rapid shrinkage of the lake in recent years^58^.

## Materials and methodology

Using the GEE platform, we mapped and monitored the trends of these climatic variables from 2000 to 2021. Additionally, a high-resolution global surface water product, available from March 16, 1984, to December 31, 2021, was integrated into the GEE platform. In the final stage of the study, the correlation coefficient between climatic variables and changes in water resources was estimated.

### Climatic variables

**Figure 3:**
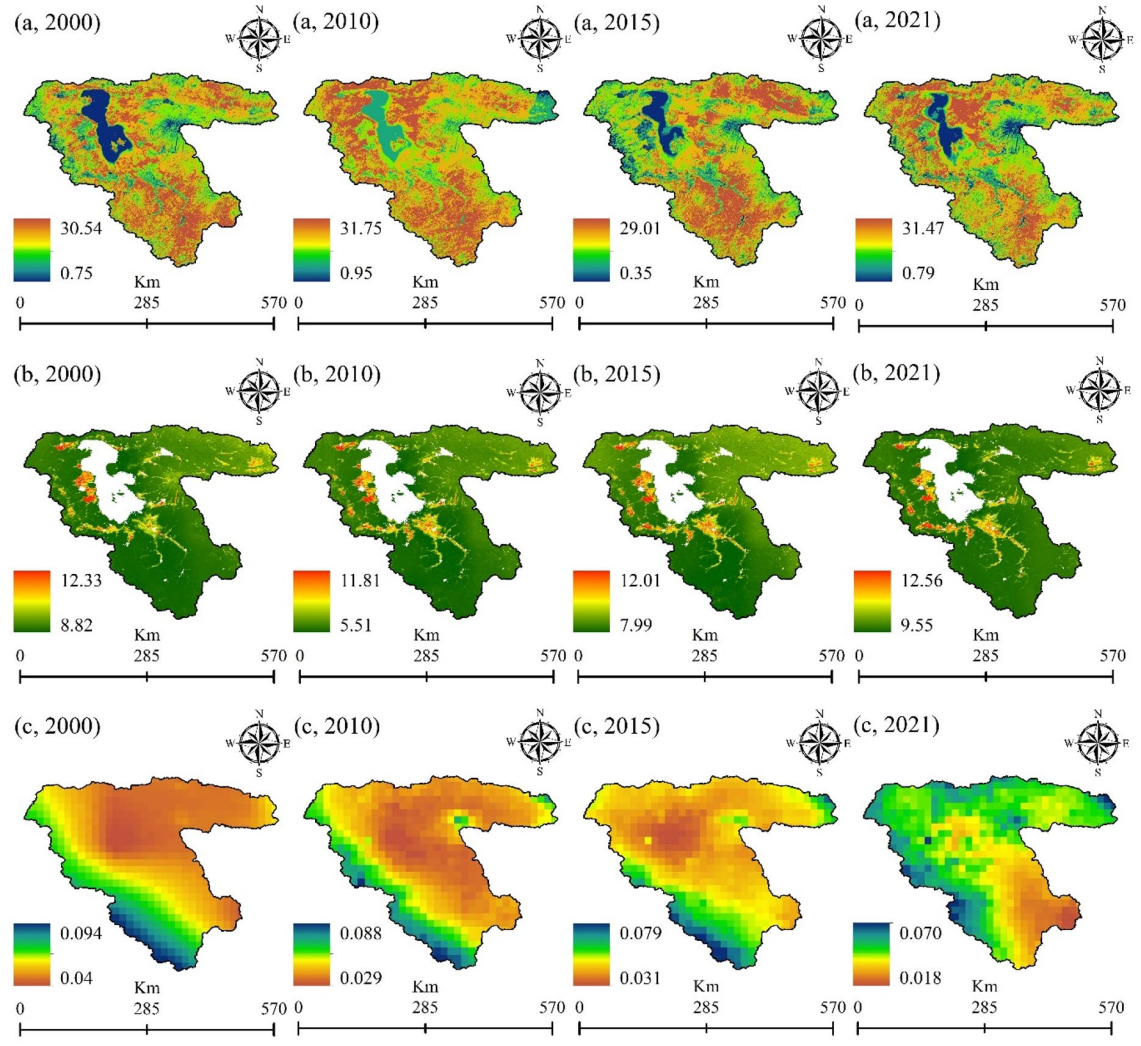
Various climatic variables for mapping and monitoring the impacts of climate change on water resources throughout the LUB, generated in the ArcGIS 10.7.1 software (www.esri.com): (**a**) Air Temperature (AT) (°C), (**b**) actual evapotranspiration (ETa) (mm/d), and (**c**) total precipitation (mm/h) for the years 2000, 2010, 2015, and 2021.

The temperature-vegetation index (TVX) method, originally introduced by Nemani et al.^59^ and further developed by Goward et al.^60^, has been successfully applied to estimate near-surface air temperature. It has been successfully applied to various satellite temperature datasets, as demonstrated by Czajkowski et al.^61^ and Lakshmi et al.^62^. In this study, we employed MODIS Land Surface Temperature (LST) products (MOD11A2.061), meteorological station data, and MODIS Normalized Difference Vegetation Index (NDVI) datasets to estimate AT using the TVX method from 2000 to 2021 (Fig. 3a). This method relies on the underlying assumption that a strong negative correlation exists between LST and a vegetation index. For more comprehensive information on the TVX method, please consult the following references:^59^ and^60^. The linear relationship between LST and NDVI is expressed by Eq. (1):

1 $$LST={a}_{t,i}+{b}_{t,i}.NDVI$$

where $t$ and $i$ represent that the regression coefficients $a$ and $b$ are time dependent and factor in each moving window $i$. Once Eq. (1) established, the AT can be extrapolated for the pixel located at the center of the moving window by allowing the linear relationship to intersect with the NDVI value associated with full vegetation cover. The estimation equation is as follows:

2 $${T}_{t,i}={a}_{t,i}+{b}_{t,i}.{NDVI}_{max}$$

where ${T}_{t,i}$ represents the AT of the pixel cantered at the moving window $i$ in time $t$, and ${NDVI}_{max}$ denotes the NDVI value of full vegetation cover.

We also used the MODIS ETa product [PML_V2 0.1.7: Coupled Evapotranspiration and Gross Primary Product (GPP)] based on the GEE platform to monitor the impacts of climate change on water resources from 2000 to 2021 (Fig. 3b). The PML_V2 0.1.7 product (unit = mm/d) provides information about 8-day evapotranspiration at a resolution of 500 m. ETa refers to the amount of moisture that evaporates from the Earth’s surface and transpires from plants to the atmosphere. As we can see in Fig. 3b, ETa rate increased about 0.32 ml over the LUB from 2000 to 2021. The maximum ETa rates were estimated to be 12.33, 11.81, 12.01, and 12.65 for the years 2000, 2010, 2015, and 2021, respectively.

Another climatic factor used in this study is the GPM: Monthly Global Precipitation Measurement (GPM) v6 product (Fig. 3c). The global precipitation measurement (GPM) is an international satellite mission that provides advanced observations of rain and snow globally every three hours. Figure 3c provides insight into the precipitation trend in the LUB between 2000 and 2021. As depicted in the figure, there has been a noticeable decrease in the precipitation rate during this time period. Specifically, the precipitation rates for the years 2000, 2010, 2015, and 2021 were estimated to be 0.094, 0.088, 0.079, and 0.070, respectively. This data underscores a clear downward trend in precipitation over the specified years.

### Water product

The JRC Global Surface Water Mapping Layers, v1.4, is a comprehensive dataset that provides detailed information about water bodies worldwide, including their extent and shoreline characteristics^36^. This dataset covers the period from 1984 to 2021 and offers maps of surface water location and temporal distribution. It offers valuable statistics regarding the extent and changes in water surfaces. Table 1 presents further details about the notable features of this product. The data is generated using 4,716,475 scenes acquired from Landsat 5, 7, and 8 between March 16, 1984, and December 31, 2021, with a spatial resolution of 30 m. An expert system is employed to classify pixels into water and non-water categories, and the results are compiled into historical periods and two epochs (1984–1999, 2000–2021) for change detection^63,64^. This mapping layer product consists of one image containing seven bands, which collectively map different aspects of surface water spatial and temporal distribution over the last 38 years. To access the JRC code via Google Earth Engine, employed in this study, use this link: https://code.earthengine.google.com/3ebfc6bab5d7ab2951020e49530df42e. Regions where water has never been detected are masked out^65^.

**Table 1:** Characteristics of the JRC Global Surface Water Mapping Layers, v1.4.

Name	Description	Min	Max	Unit
Occurrence	The frequency with which water was present	0	100	%
Change absolute	Absolute change in occurrence between two epochs: 1984–1999 and 2000–2021	− 100	100	%
Change_norm	Normalized change in occurrence. (epoch1-epoch2)/(epoch1 + epoch2) * 100	− 100	100	%
Seasonality	Number of months, water is present	0	12	–
Recurrence	The frequency with which water returns from year to year	0	100	%
Max_extent	Binary image containing 1 anywhere water has ever been detected	–	–	–
Transition	Categorical classification of change between first and last year	–	–	–

### Methodology

**Figure 4:**
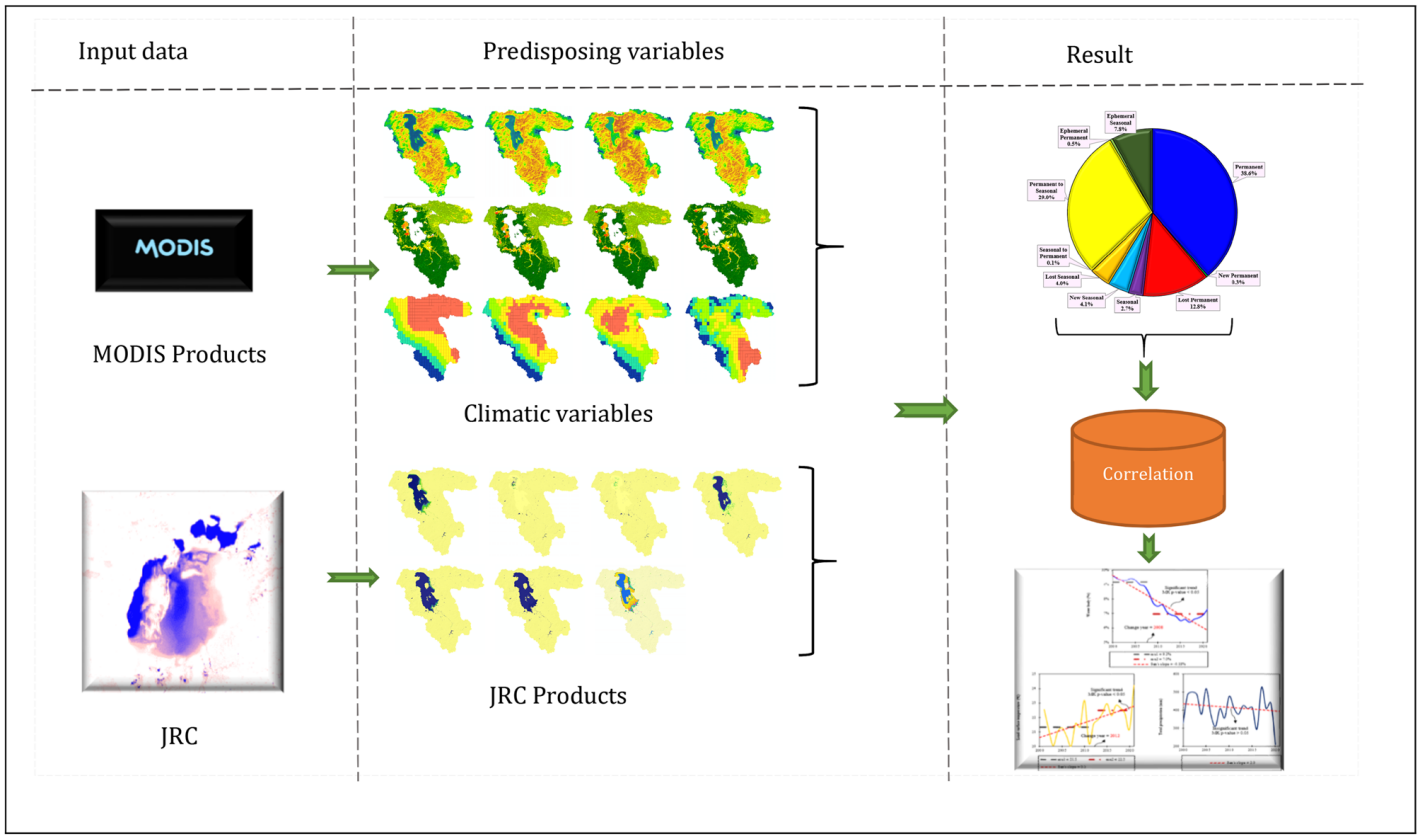
A brief review of the applied methodology for mapping and monitoring the impacts of climate change on water resources, generated in the Microsoft Office Word 2023 (https://www.microsoft.com).

This study employed an integrated approach that combined remote sensing techniques and the GEE to monitor the effects of climate change on water resources in the LUB. Figure 4 provides an overview of the methodology used for mapping and monitoring the impacts of climate change on water resources.

### Google Earth Engine

The GEE offers a wide range of geospatial datasets and satellite imagery, accompanied by powerful spatial analysis capabilities. It is extensively used by researchers, scientists, and industry professionals to generate maps that facilitate the detection of surface changes on Earth. In the context of this study, various MODIS and Landsat global water surface products were employed within the GEE platform. Data retrieval on the GEE platform is straightforward, requiring only a few commands without the need to handle data loading manually. The platform automatically processes the commands and manages the data retrieval process^66,67^. Since the command takes care of calling the dataset, radiometric and geometric correction are not necessary. Users are expected to write code according to their specific processing requirements. The implemented code facilitated the execution of the following operations.

- Spatial subset on the interested area;
- Temporal subset on the collection datasets;
- Selecting the targeted datasets;
- Exporting the selected datasets to perform further analysis.

### Statistical analysis

The Mann–Kendall (MK) non-parametric trend test^68^ was employed to identify statistically significant trends (*p* < 0.05) in the time series data (Eqs. 3 and 4). To address serial correlation effect, the modified version of the MK test proposed by^69^ was applied. Moreover, the Spearman partial correlation analysis^70^ was conducted to determine the most influential climate factor contributing to the dynamics of water resources. These statistical techniques have been widely employed in previous studies investigating climate variability and change in water resources worldwide (e.g.,^71^).

3 $$S=\sum_{i=1}^{n-1}\sum_{j=i+1}^{n}sgn({x}_{j}-{x}_{i})$$

4 $$sgn\left({x}_{j}-{x}_{i}\right)=\left\{\begin{array}{c}+1\\ 0\\ -1\end{array} \begin{array}{c}{x}_{j}>{x}_{i}\\ {x}_{i}={x}_{j}\\ {x}_{j}<{x}_{i}\end{array}\right.$$

where $n$ represents the length of the time series, and ${x}_{i}$ and ${x}_{j}$ denote the sequential data amounts, respectively. For $n>10$, given that ${x}_{i}$ is independent and randomly ordered, the statistic $S$ is approximately normally distributed with a mean of zero.

## Results

**Figure 5:**
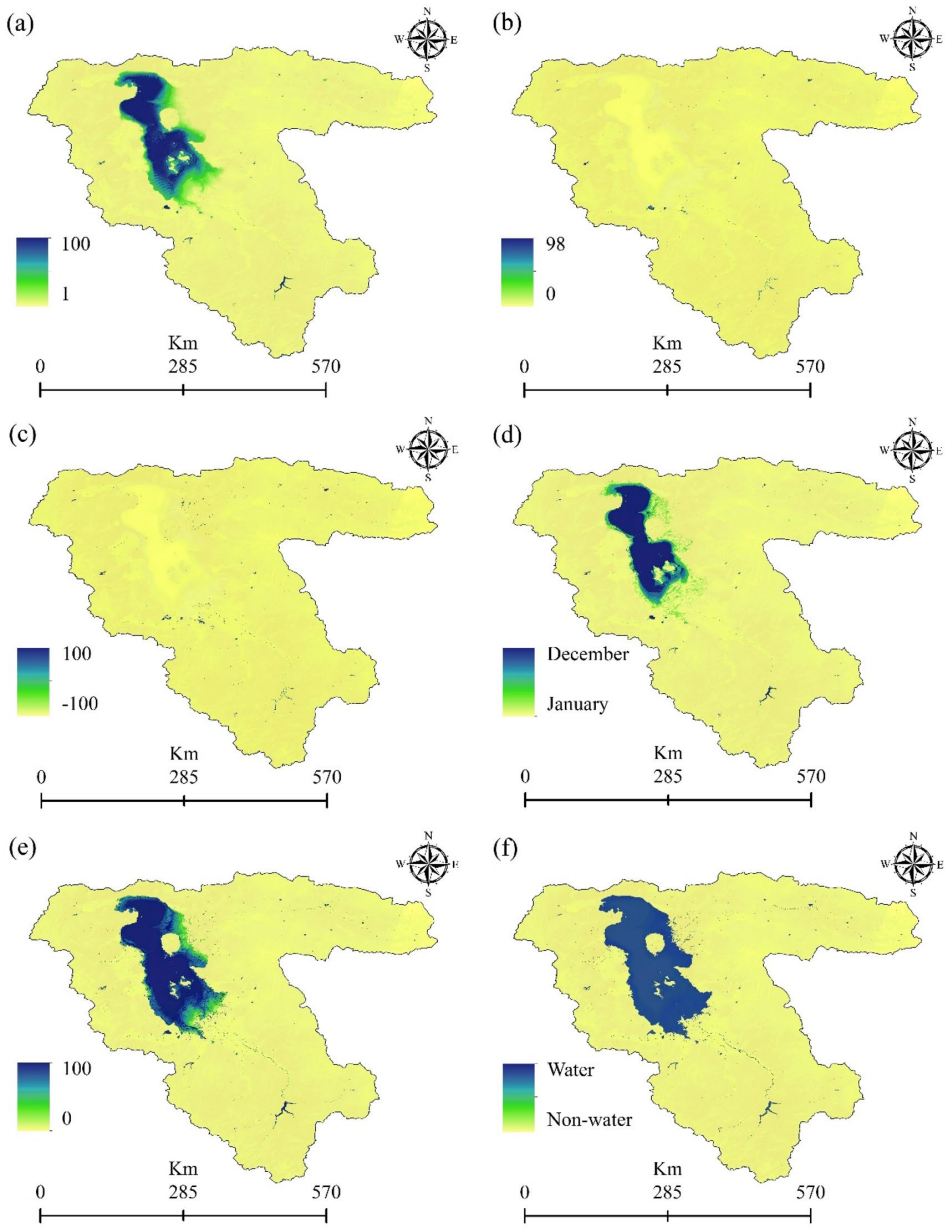
Results of the employed Landsat high-resolution global water surface product, generated in the ArcGIS 10.7.1 software (www.esri.com): (**a**) frequency of water presence, (**b**) absolute change in occurrence between 1984 and 2021, (**c**) normalized change in occurrence between 1984 and 2021, (**d**) number of months with water presence, (**e**) frequency of water return from year to year, and (**f**) water detection indicator.

**Figure 6:**
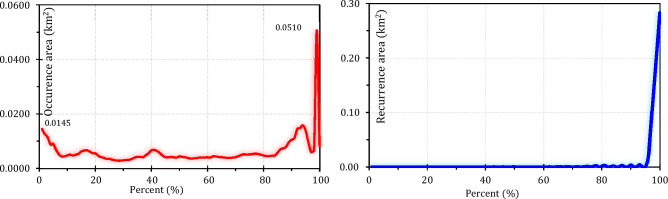
Frequency of water body occurrence in the LUB from 1984 to 2021. Recurrence frequency of water body from year to year in the LUB from 1984 to 2021, generated in the Microsoft Office Excel 2023 (https://www.microsoft.com).

**Figure 7:**
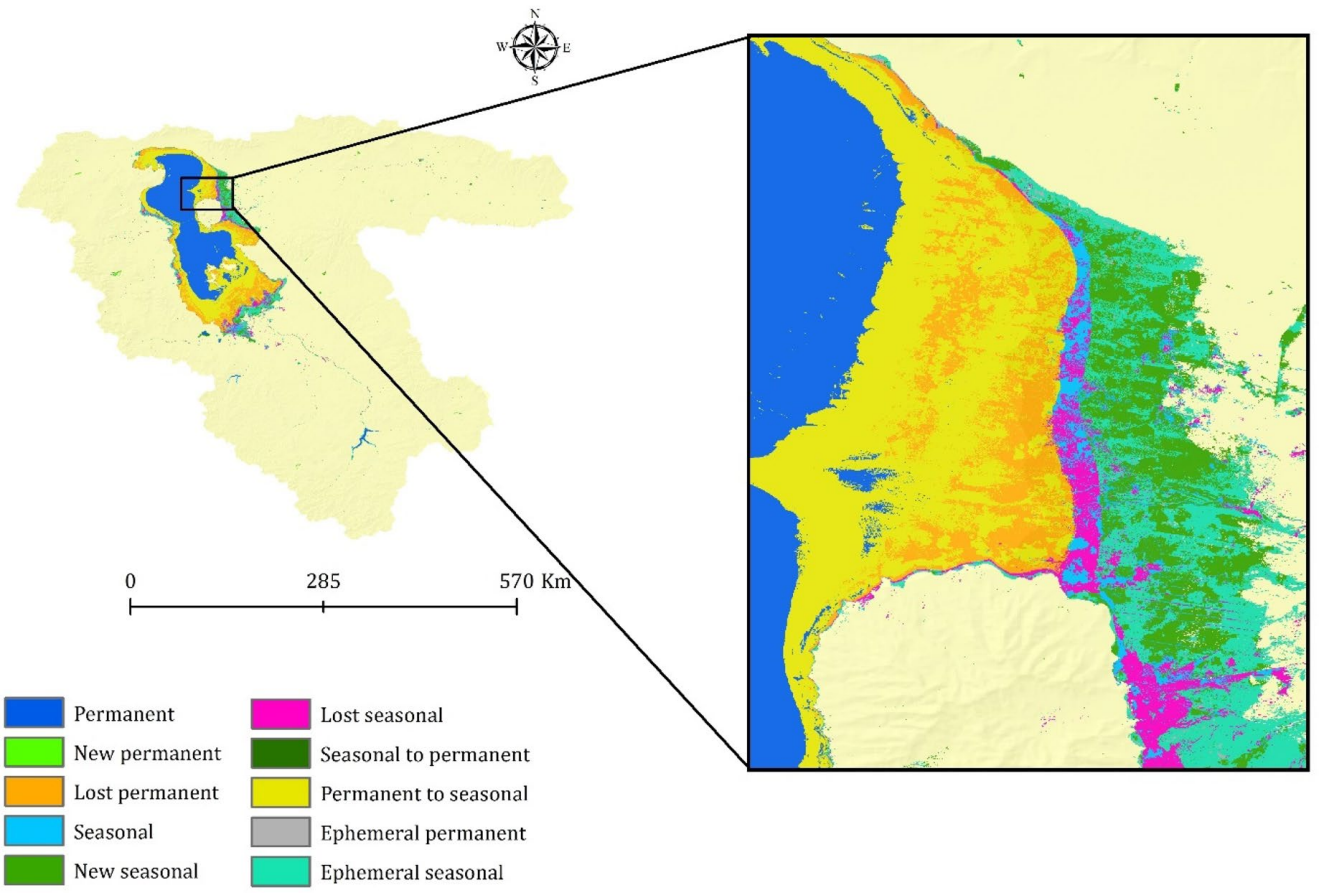
Transitions in water bodies across the LUB from the first year (1984) to the last year (2021), generated in the ArcGIS 10.7.1 software (www.esri.com).

**Figure 8:**
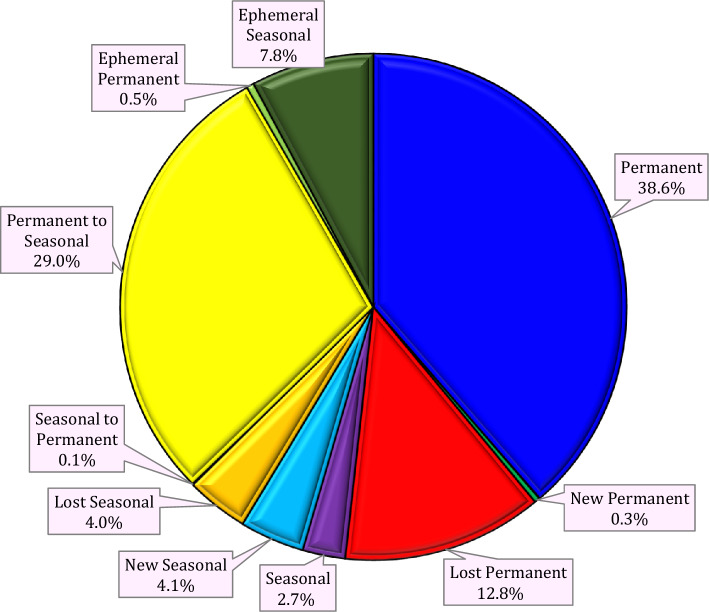
Changes in water body transitions from 1984 to 2021 in the LUB, generated in the Microsoft Office Excel 2023 (https://www.microsoft.com).

This study applied an integrated approach involving remote sensing datasets and GEE to monitor climate change effects on water resources over the LUB, Iran. Figures 5 and 7 show the results of water surface changes over the study area. Based on Fig. 5a, which illustrates the frequency of water presence from 1984 to 2021 (see Table 2), it is evident that in the central parts of the LU, water has consistently been present throughout this period, accounting for 0.99% of the area. This corresponds to an approximate area of 0.051 km^2^ (as shown in Figure 6). In contrast, the surrounding areas of the central parts have undergone significant changes in terms of water presence. Figure 5b provides insight into the absolute change in water occurrence between two epochs: 1984–1999 and 2000–2021 (see Table 2). The findings reveal that there has been a significant change in water presence across most of the LUB area. With the exception of dams and a small portion of the LU in the northern part of the LUB, the entire area has experienced such changes from 1984 to 2021. According to Table 2, approximately 73.58% of the study area has undergone an absolute change in water presence during the period of 1984–2021. Similarly, apart from dams and a small part of the LU in the northern region of the LUB, there has been a substantial change in surface water presence from 1984 to 2021, which illustrates the normalized occurrence change (see Fig. 5c). Table 3 further supports this observation, indicating that approximately 66.29% of the study area within the LUB has experienced a change in water presence over the period of 1984–2021. Figure 5d provides insights into the number of months during which water was present in the study area from 1984 to 2021. The data presented in Fig. 5d and Table 4, indicate that January (10.82%) and December (56.08%) have a high level of water availability. The high water availability in December and January can be attributed to the occurrence of snow, which is common during the winter season in the study area. Furthermore, the increase in water surface during the spring months of April, May, and June can be attributed to the higher amount of precipitation in the study area during this season. Figure 5e in this study displays the frequency of water return from year to year between 1984 and 2021. It indicates that the central parts of the LU have the highest percentage of water return, suggesting that these areas have experienced more consistent recovery of water over time. On the other hand, the surrounding areas of the LU, as depicted in Fig. 5e, have not witnessed significant water recovery from year to year (Fig. 6). Figure 7 presents a categorical classification of the changes that occurred between the first and last years from 1984 to 2021. According to Fig. 7, the surrounding areas of the LU have undergone significant changes in terms of water conditions compared to previous years. Out of the total, 38.65% has been identified as permanently water-covered from 1984 to 2021. Within this permanent water area, 0.34% has been newly classified as permanent, as indicated in Fig. 8. Over the study period, a permanent water area of 12.78% has been lost. Additionally, 2.74% has been detected as seasonal water resources from 1984 to 2021. While 3.98% of the LUB has experienced a loss in seasonal water resources, 4.12% has been newly added as seasonal water resources, as illustrated in Fig. 8. Figure 8 shows that there has been no change in the conversion of seasonal to permanent water resources over the study area from 1984 to 2021. However, 28.99% of the permanent water has been converted to seasonal water in the LUB during this period, as depicted in Fig. 8. Furthermore Fig. 8 indicates that 0.48% and 7.84% of the water resources have been classified as ephemeral permanent and ephemeral seasonal, respectively, over the study area from 1984 to 2021.

**Table 2:** Absolute change in water occurrence in the LUB from 1984 to 2021.

Class	Area (km^2^)	Area (%)
1	0.003021	41.07
2	0.001367	18.59
3	0.001024	13.92
4	0.000525	7.14
5	0.000975	13.26
6	0.000168	2.28
7	0.000211	2.87
15	0.000007	0.10
16	0.000009	0.12
17	0.000002	0.03
18	0.000015	0.20
19	0.000011	0.15
20	0.000005	0.07
25	0.000005	0.07
32	0.000001	0.01
40	0.000001	0.01
55	0.000002	0.03
65	0.000002	0.03
70	0.000001	0.01
86	0.000002	0.03
90	0.000001	0.01

**Table 3:** Normal change in water occurrence in the LUB from 1984 to 2021.

Class	Area (km^2^)	Area (%)
1	0.003882	33.92
2	0.001173	10.25
3	0.002532	22.12
4	0.000522	4.56
5	0.000270	2.36
6	0.000002	0.02
9	0.000001	0.01
10	0.000004	0.03
11	0.000006	0.05
13	0.000003	0.03
14	0.000010	0.09
16	0.000013	0.11
17	0.000024	0.21
18	0.000003	0.03
25	0.000001	0.01
33	0.000008	0.07
34	0.000002	0.02
35	0.000014	0.12
42	0.000002	0.02
45	0.000003	0.03
60	0.000002	0.02
66	0.000003	0.03
89	0.000000	0.00
92	0.000002	0.02
93	0.000007	0.06
95	0.000001	0.01
97	0.000007	0.06
100	0.002949	25.76

**Table 4:** Water body presence in each month in the LUB from 1984 to 2021.

Months	Area (km^2^)	Area (%)
Jan	0.046842	10.82
Feb	0.015637	3.61
March	0.007076	1.63
April	0.016594	3.83
May	0.022464	5.19
Jun	0.018861	4.36
July	0.021056	4.86
August	0.006011	1.39
September	0.005988	1.38
Oct	0.005968	1.38
November	0.02368	5.47
Dec	0.242827	56.08

**Figure 9:**
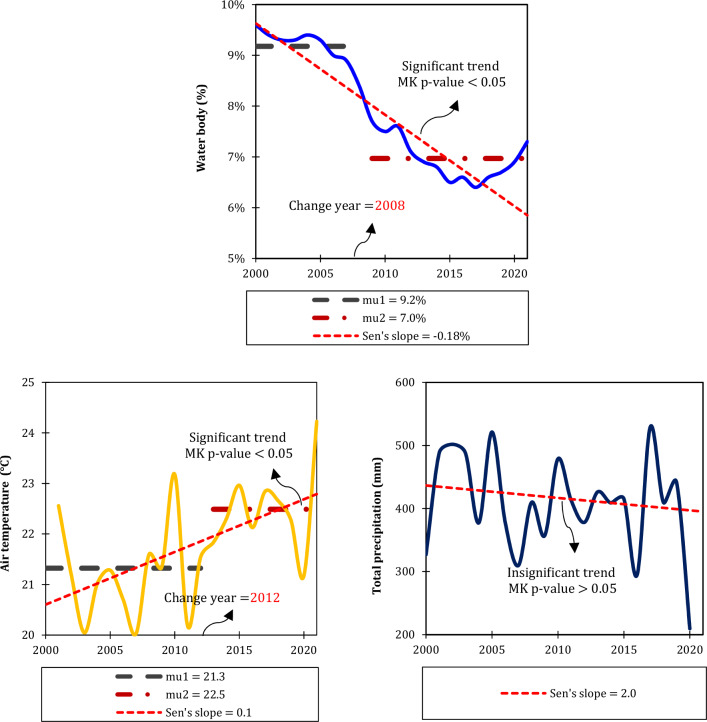
Time series analysis depicting the annual variations and trends in surface water bodies and climatic variables including air temperature (AT) and total precipitation, throughout the LUB, generated in the Microsoft Office Excel 2023 (https://www.microsoft.com). The significance of the trend lines was determined using the Mann–Kendall test. The trend line slope is based on Sen's slope estimator. In cases where a time series experienced a significant abrupt shift, identified by Buishand's test, the change year is indicated. For these instances, “mu” denotes the average of the sub-series.

Our findings indicate correlation coefficients of − 0.55, − 0.59, and 0.39 between water area and AT, ETa, and precipitation, respectively (Fig. 9 and Table 5). According to our research, climate change has affected water resources from 1984 to 2021, which is in accordance with the results of^22^. They observed that over the past few decades, a warming trend of approximately 0.18 °C per decade has been identified, while precipitation in the basin has been decreasing by about 9 mm per decade. As a result of this significant warming, ETa from the lake has been increasing at a rate of 6.2 mm per decade. The rising AT and increased ETa, combined with the decreasing precipitation, indicate that LUB has been experiencing meteorological drought conditions. In addition to climate change effects^19^, discovered a roughly 98% increase in agricultural lands and a substantial 180% increase in urban areas between 1987 and 2016. Consequently, the lake area experienced a significant reduction of around 86%. Their studies also revealed a decline in terrestrial water storage in the lake region, with the lake losing water at a faster rate than the watershed. Comparing river inflow to the lake, it is evident that human water management activities resulted in a reduction in streamflow of approximately 1.74 km^3^/year from 1995 to 2010. This reduction accounts for approximately 86% of the total decrease in the lake's volume during the same period^72^ also indicated that the process of change in LU was slow between 1970 and 1997. However, the shrinkage accelerated between 1998 and 2018, with approximately 30.00% of the lake's area disappearing. According to their findings, anthropogenic factors, such as population growth, extensive dam construction, low irrigation water use efficiency, poor water resources management, increased sediment flow into the LU, and the absence of political and legal frameworks, had a much greater impact on LU than climate change and prolonged drought. The mismanagement of water consumption in the agricultural sector and the extraction of surface and groundwater from the basin have resulted in a sharp decrease in the lake's surface area.

**Table 5:** Spearman correlations and partial correlations of the water surface area with climatic variables, including AT and total precipitation, throughout the LUB over the period 2000–2021.

	Spearman correlation		Spearman partial correlation	
	AT	Precipitation	AT	Precipitation
Water surface	**-*0.609***	0.005	***-0.656***	0.258

Statistically significant correlations and partial correlations (**p** < 0.05) are presented in both bold and italic font and coloured in red.

In this study, the water surface area in the LUB exhibited a statistically significant (*p* < 0.05) decline of 2.0% decade^−1^ over the past two decades. This decline was accompanied by a significant downward abrupt shift in 2008, resulting in an average sub-series of 9% before the shift and 7% after the shift. Conversely, AT showed a significant increase of 1.02 °C decade^−1^, with an upward abrupt shift identified in 2012. The average sub-series after the shift was 1.2 °C higher than the average before the shift. On the other hand, annual total precipitation experienced a decline of 20 mm decade^−1^ in the LUB during the period of 2000–2021, but no statistically significant trends or variations were observed. In general, there were negative correlations observed between water surface and AT, while positive correlations were found with total precipitation throughout LUB from 2000 to 2021 (Table 5). It was observed that an increase in AT corresponded to a decrease in water surface area, highlighting the significant influence of AT and ETa on controlling the water surface in the LUB (partial rho of − 0.65 and − 0.68, respectively) (See^22^). A partial correlation coefficient assesses the strength and direction of a relationship between two variables while controlling for the influence of other variables. In this case, it indicates a moderately strong negative correlation between AT and water surface area when considering the effects of other potential influencing factors. These findings imply that as AT rises, there is a tendency for water surface area to decrease. It is important to note that correlation does not necessarily imply causation, and further analysis might be needed to establish the underlying mechanisms driving this relationship. In practical terms, these findings could have implications for understanding and managing water resources in semi-arid and arid regions. High AT might lead to increased ET or changes in hydrological patterns, which could impact water availability and ecosystem dynamics. Conversely, no significant relationship was found with precipitation and water surface area (partial rho of + 0.25). The partial correlation coefficient of + 0.25 suggests a weak positive correlation between these two variables when accounting for other factors. This means that, in the context of our study, changes in precipitation do not seem to have a strong influence on variations in water surface area. This finding aligns with the work of^54^, who also reported a lack of significant correlation between water level and precipitation in the case of the LU. In their study, they demonstrated a significant negative correlation (rho of − 0.68) between water level and temperature, indicating that as temperature increased, the water level decreased. However, they found no significant correlation (rho of + 0.10) between water level and precipitation. These consistent findings across different studies highlight the complex interactions between climatic variables and water bodies. While temperature appears to have a more pronounced impact on water surface area or water levels, precipitation might not exhibit a clear relationship in certain contexts. This could be due to the influence of various other factors, such as ET rates, runoff patterns, and local hydrological characteristics. The results of the partial correlation analysis, which aimed to identify the most influential climate factor contributing to water resource dynamics while considering the effect of each studied climatic variable, revealed a statistically significant (*p* < 0.05) correlation of − 0.656 between water surface and AT. However, no significant correlation was found between water surface and precipitation, indicating that temperature plays a dominant role in controlling water surface in the LUB.

## Discussion

This study was applied an integrated approach of remote sensing and GEE for monitoring the effects of climate change on water resources. The LUB was selected as the case study area, which has experienced significant changes in water resources in the last decades. The results of this study provide valuable insights into the impact of climate change on water resources in the LUB from 1984 to 2021. This study reveals a statistically significant change in the water surface area over the past two decades. This suggests that there has been a notable alteration in the extent of water bodies in the LUB during this period like previous studies^23^. AT and ETa in the LUB have shown significant increases from 2000 to 2021 aligns with the findings of numerous studies on climate change and land use patterns^73,74^. These increases in AT and ETa are indicative of warming in the region, which is a common consequence of climate change. In contrast to the rising AT and ETa, the study observes a decline in annual total precipitation in the LUB during the period from 2000 to 2021. The reduction in precipitation observed over the study area, as indicated by previous studies^73,75^, is consistent with broader climate change projections and observations in many regions globally. This suggests that the region has been experiencing reduced rainfall or other changes in precipitation patterns. The research indicates negative correlations between water surface and AT and ETa. This means that as AT and ETa increase, there is a tendency for the water surface area to decrease. This relationship aligns with the expectation that warmer temperatures can lead to evaporation and reduced water availability. Conversely, there are positive correlations between water surface and total precipitation. This implies that when there is more precipitation, there tends to be a larger water surface area. This relationship underscores the importance of precipitation in maintaining water resources. In summary, the research findings suggest that climate change has led to increase AT and ETa and reduce annual total precipitation in the LUB from 2000 to 2021. These changes have had a noticeable impact on water resources, as evidenced by the negative correlation between AT, ETa and water surface area.

This study also was employed a data-driven approach by utilizing the automatic product called JRC-Global surface water mapping layers V1.4 on the GEE platform for monitoring water surface area changes. Previous studies (e.g.,^48,72^) have relied on traditional or semi-automatic methods, which typically provide an accuracy of around 500 m. However, in the current study, a more accurate approach was employed. This approach offers a significantly improved accuracy of 30 m, allowing for more precise monitoring and mapping of all water bodies, including seasonal and small rivers. By leveraging this higher accuracy, the study was able to reveal important findings. The JRC Global Surface Water Mapping Layers, version 1.4, provides valuable data on the extent and changes of water surfaces. In contrast to GLAD, GSWD, and DSWE, this dataset offers comprehensive statistics regarding various aspects of water bodies, including seasonal fluctuations, temporal alterations, monthly variations, and details on permanent reservoirs. Each of these datasets has its unique characteristics and time coverage, but the JRC Global Surface Water Mapping Layers, v1.4, is particularly noteworthy for its comprehensive insights and up-to-date information on water surfaces. The results of the study indicated that over the past 4 decades, only approximately 40% of the water bodies in the LUB remained permanent. This suggests a loss of around 30% of the permanent water resources, which have transitioned into seasonal water bodies, accounting for nearly 13% of the total. These findings highlight the significant changes that have occurred in the water resources of the basin and emphasize the importance of accurate and detailed monitoring methods in assessing and understanding such changes.

Water scarcity in the LUB causes several ecological problems. Different studies have proven that the shrinking the lake resulted in a 90% decline in the Artemia population^76^. Moreover, the salinity has dramatically increased in recent years, reaching a point where the lake water is saturated with salts and salt crystals form on the lake surface throughout the year. This has also led to the conversion of surrounding land into salt marshes. A recent study by Sima et al.^77^ reported that the flamingo population in the lake has decreased to almost zero. The loss of water resources in the LUB has resulted in a general decrease in vegetation, leading to a dramatic increase in the frequency and intensity of dust storms in the region. Dust storms have a detrimental impact on over 7 million residents in the region, causing various challenges related to health, socioeconomic conditions, soil fertility in agriculture, and vegetation weakening. These issues present significant concerns that need to be addressed to safeguard the well-being of the affected population and maintain the ecological balance of the region. Furthermore, frequent decreases in the volume of water resources would affect food security in the study area. It is because agriculture over the LUB is the main source of food, and water scarcity would pose a significant threat to this sector^78^. In summary, as the consequence of climate change intensify and the water crisis in the basin worsens, the region may face a significant challenge in terms of migration from rural areas to suburban areas of metropolitan cities. This migration crisis, along with its accompanying socio-economic costs, will pose a major obstacle to regional development.

### Limitation of the study and future perspective

The main limitation of this study is related to the temporal variation of the JRC Global Surface Water Mapping Layers, v1.4 product. Since there is no information related to water surface storage for each year separately, having information for each year separately that shows all various aspects of water surfaces, including seasonal, temporal, monthly, and permanent reservoir information, is of great importance. This provides valuable information for monitoring changes in water patterns.

## Conclusion

The application of remote sensing in studying the impacts of climate change on water scarcity necessitates effective, efficient, and cost-effective technologies capable of analyzing large datasets. Therefore, this study was initiated to map various influential climatic variables and determine the impacts of climate change on water resources. The findings of this study indicate correlation coefficients of − 0.55, − 0.59, and 0.39 between water area and AT, ETa, and precipitation, respectively. According to the results, climate change has affected water resources from 1984 to 2021 in the LUB. The results also demonstrate that a permanent water area of 12.78% was lost from 1984 to 2021 in the LUB. Additionally, 2.74% has identified as seasonal water resources. While 3.98% of the LUB has experienced a decline in seasonal water resources, an additional 4.12% has newly added as seasonal water resources. A general monitoring framework was applied to analyze climate feature changes across extensive regions at different temporal scales. The study further reveals that online-based approaches, such as GEE combined with remote sensing datasets, are valuable for monitoring climate change impacts, particularly in semi-arid and arid regions, to simulate dynamic climate changes. In summary, the results of this study hold great significance for water management planners and managers and contribute significantly to the advancement of GIS science through the application and identification of effective techniques for mapping climate change effects on water resources.

## Data Availability

The datasets used and/or analysed during the current study available from the corresponding author on reasonable request.
